# Supplementary material for: DNA methylation is reconfigured at the onset of reproduction in rice shoot apical meristem
Source: Nat Commun. 2020 Aug 14;11:4079. doi: 10.1038/s41467-020-17963-2 (PMC7429860; doi:10.1038/s41467-020-17963-2)
Supplement: Supplementary file 1 — Supplementary Information [file 41467_2020_17963_MOESM1_ESM.pdf]

Supplementary Information

DNA methylation is reconfigured at the onset of reproduction in rice shoot apical meristem

Higo et al.

Contents

Supplementary Table 1
Supplementary Figures 1 - 14

Supplementary Table 1. Summary of re-sequencing of N8 genome, BS-seq, and smRNA.

N8 genome resequencing

DNA amounts	Total read counts	Mapped reads	Mapping rate	Average depth
1 µg	319,255,142	307,816,941	96.4%	80.2

BS-seq (leaf, vegetative SAM, and reproductive SAM)

Sample name	Number of SAM	DNA amounts	Total read counts	Mapped reads	Mapping rate	Coverage of cytosines	Average depth
Vegetative SAM	164	35.8 ng	381,703,490	196,292,010	51.4%	85.3%	25.2
Reproductive SAM	140	32.8 ng	373,479,109	191,989,131	51.4%	85.2%	24.6
Mature leaf blade	-	100 ng	311,872,808	152,822,192	49.0%	84.5%	19.6

smRNA-seq

Sample name	Total read counts
Vegetative SAM	57,304,871
Reproductive SAM	48,341,898
Mature leaf blade	37,511,914

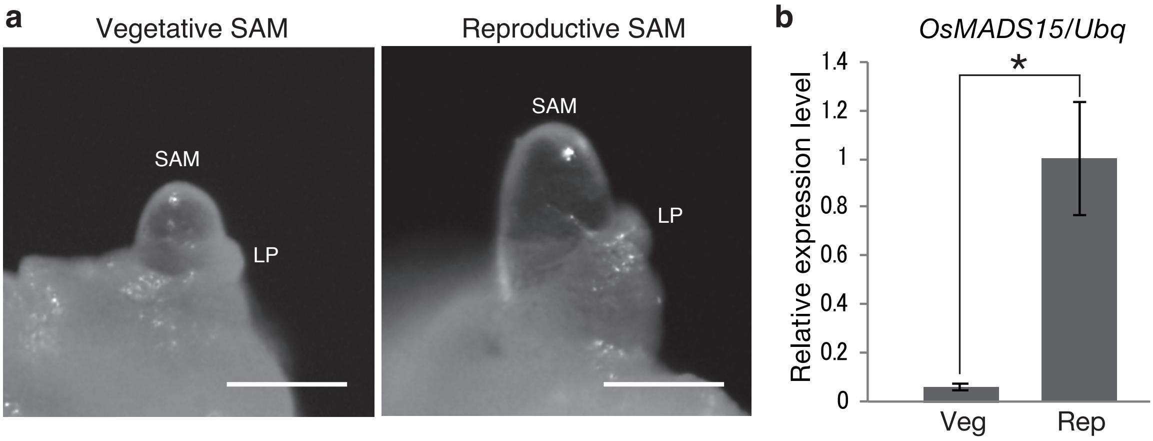

Supplementary Fig. 1. Vegetative and reproductive SAMs of rice. a, Examples of rice vegetative (right) and reproductive SAMs (left). After the floral transition, the SAM elongates and enlarges. Bars = 100 µm. LP, leaf primodia. **b,** Relative expression level of *OsMADS15*, which is expressed at the beginning of the reproductive stage in vegetative (Veg) and reproductive (Rep) SAMs. *OsMADS15* expression in the reproductive SAM was set to 1. *Ubq* was used as the internal control. Error bars indicate mean ± SD; $n = 3$ (*$p<0.01$; Student's *t*-test).

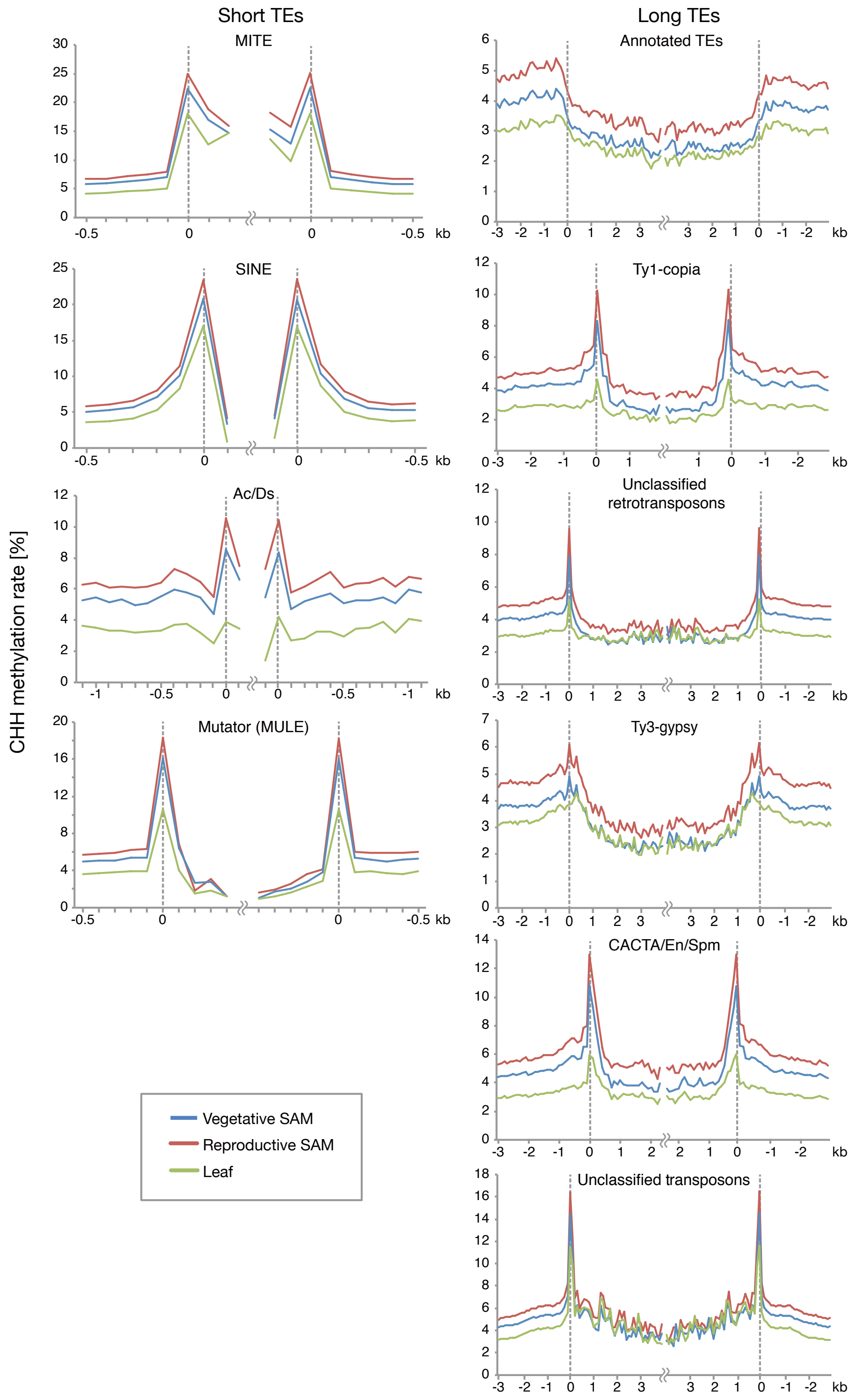

Supplementary Fig. 2. CHH methylation patterns in various TE families. Average CHH methylation rates within 100-bp windows in vegetative SAM (blue), reproductive SAM (red), and mature leaf blades (green) were plotted around TE bodies of the indicated families. Vertical broken lines represent the boundaries of the TE body.

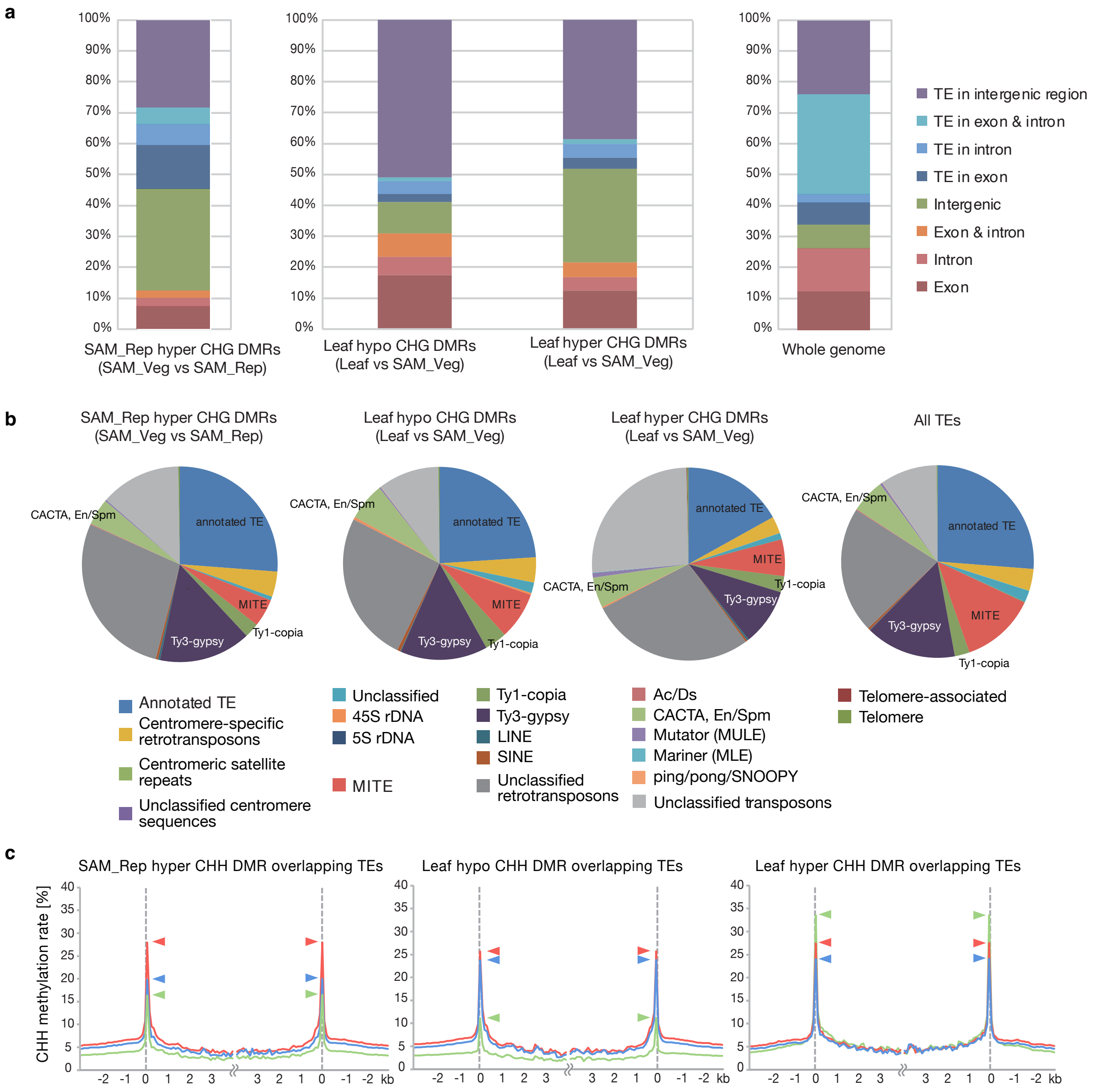

Supplementary Fig. 3. Features of DMRs. a, Genomic distribution of SAM Rep-hyper CHG DMRs (left, SAM_Veg vs SAM_Rep), Leaf hypo CHG DMRs (middle, Leaf vs SAM_Veg), and hyper CHG DMRs (right, Leaf vs SAM_Veg). Genomic distribution of TEs in the genomic features are shown as whole genome. **b,** TE families overlapping with Rep-hyper CHG DMRs between vegetative and reproductive SAM (left, SAM_Veg vs SAM_Rep), Leaf hypo CHG DMRs (middle, Leaf vs SAM_Veg), and hyper CHG DMRs (middle, Leaf vs SAM_Veg). Ratio of length for each TE family are shown as All TEs. **c,** MetaTE plots for TEs overlapping with DMRs. Plots show the CHH methylation patterns around TEs in mature leaf blade (green), vegetative SAM (blue), and reproductive SAM (red). TEs were aligned at the 5′ end or the 3′

end and average CHH methylation level was plotted. Methylation level within each 100-bp interval was averaged and plotted from 3 kb away from TEs (negative numbers) to 4 kb into the annotated regions (positive numbers). Dashed lines represent the points of alignment. Arrowheads indicate the CHH methylation peaks at the edges of the TE body.

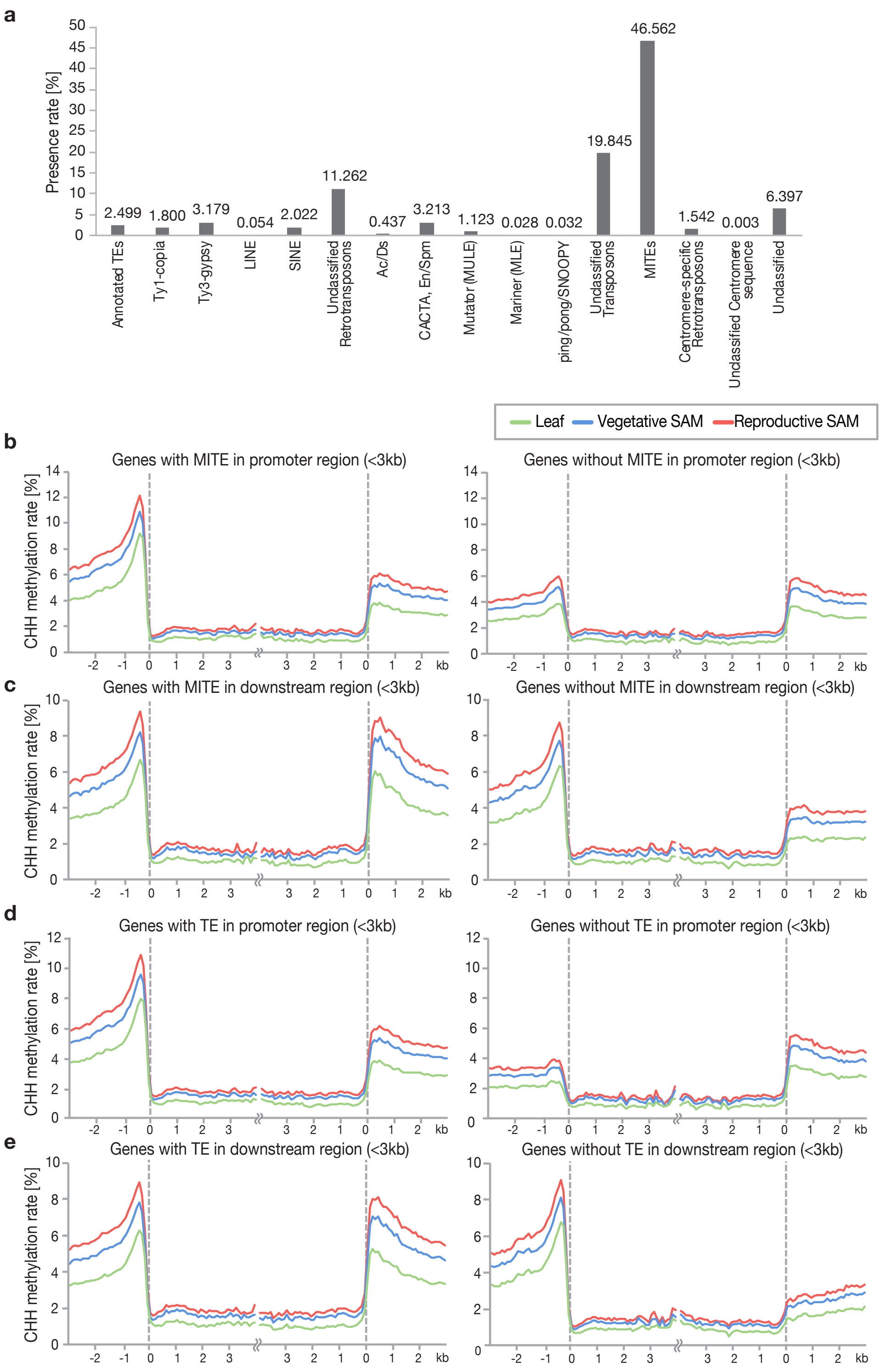

Supplementary Fig. 4. The effect of TE insertion on CHH methylation pattern around protein-coding genes. a, Occupancy of each TE family in promoter regions of rice genes (< 3 kb upstream of protein-coding genes in the genome). **b-e,** Metagene plots show patterns of DNA methylation for the CHH context in mature leaf blade (green), vegetative

SAM (blue), and reproductive SAM (red) around protein-coding genes with (left) or without (right) MITE (b, c) or all TEs (d, e) in their upstream (b, d) and downstream (c, e) regions. Protein-coding genes were aligned at the 5′ end or the 3′ end and average methylation level for the CHH context was plotted. Methylation level within each 100-bp interval was averaged and plotted from 3 kb away from the protein-coding genes to 4 kb into the annotated regions. Dashed lines represent the points of alignment.

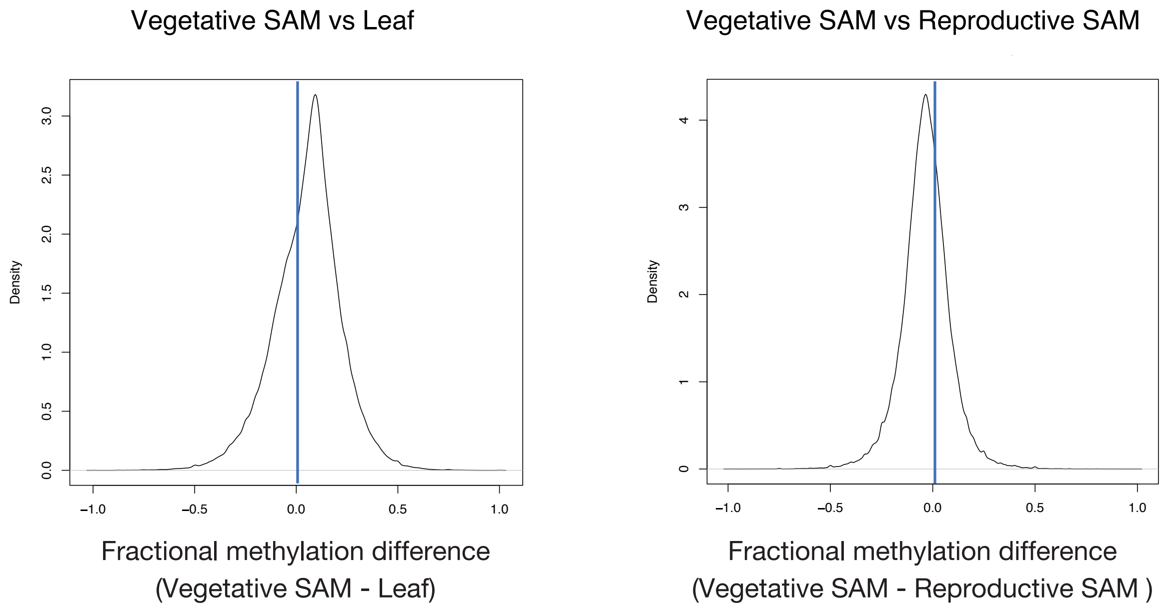

Supplementary Fig. 5. Methylation difference among leaf, vegetative SAM, and reproductive SAM. Density plots show the frequency distribution of the methylation differences within 50-bp windows of the MITEs in the rice genome between vegetative SAM and mature leaf blade (left) and vegetative SAM and reproductive SAM (right).

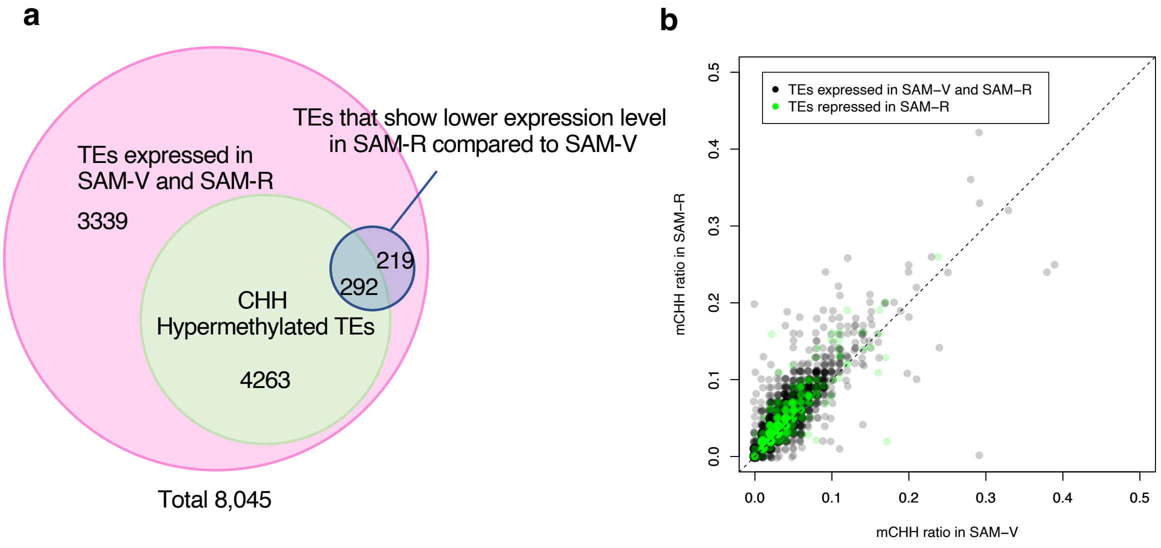

Supplementary Fig. 6. CHH hypermethylation is globally increased in TEs

a, Venn diagram showing overlap among expressed TEs, mCHH hypermethylated TEs and silenced TEs. **b,** Scatter plot showing CHH methylation rates. Each dot represents the average methylation rate within TEs expressed in SAM-V and SAM-R (black) and TEs repressed in SAM-R (green). Dotted line shows y = x axis.

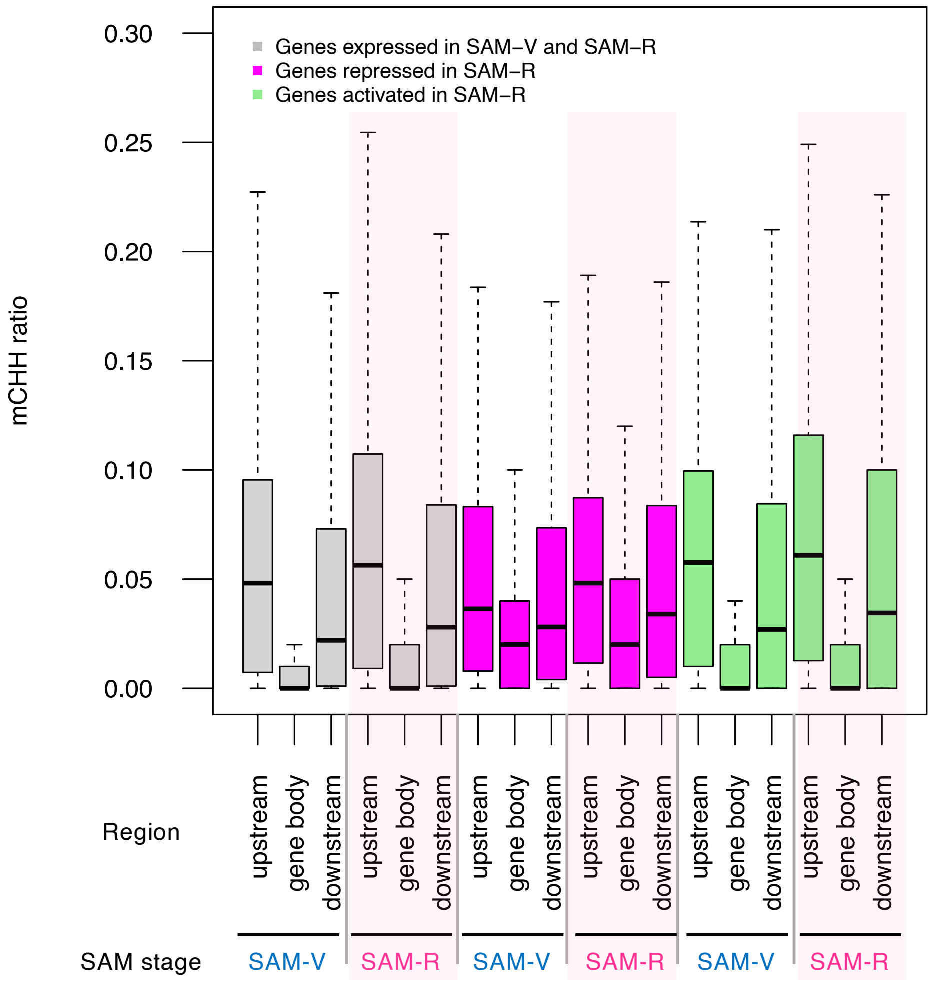

Supplementary Fig. 7. CHH hypermethylation is globally increased in genes. Boxplot shows ratio of CHH methylation (mCHH) in SAM-V and SAM-R for genes expressed in SAM-V and SAM-R (gray), repressed in SAM-R (magenta), and activated in SAM-R (green). mCHH ratios for regions from 1 kb upstream to the beginning of annotation (upstream), gene body, and regions from end of annotation to 1 kb downstream (downstream) are indicated.

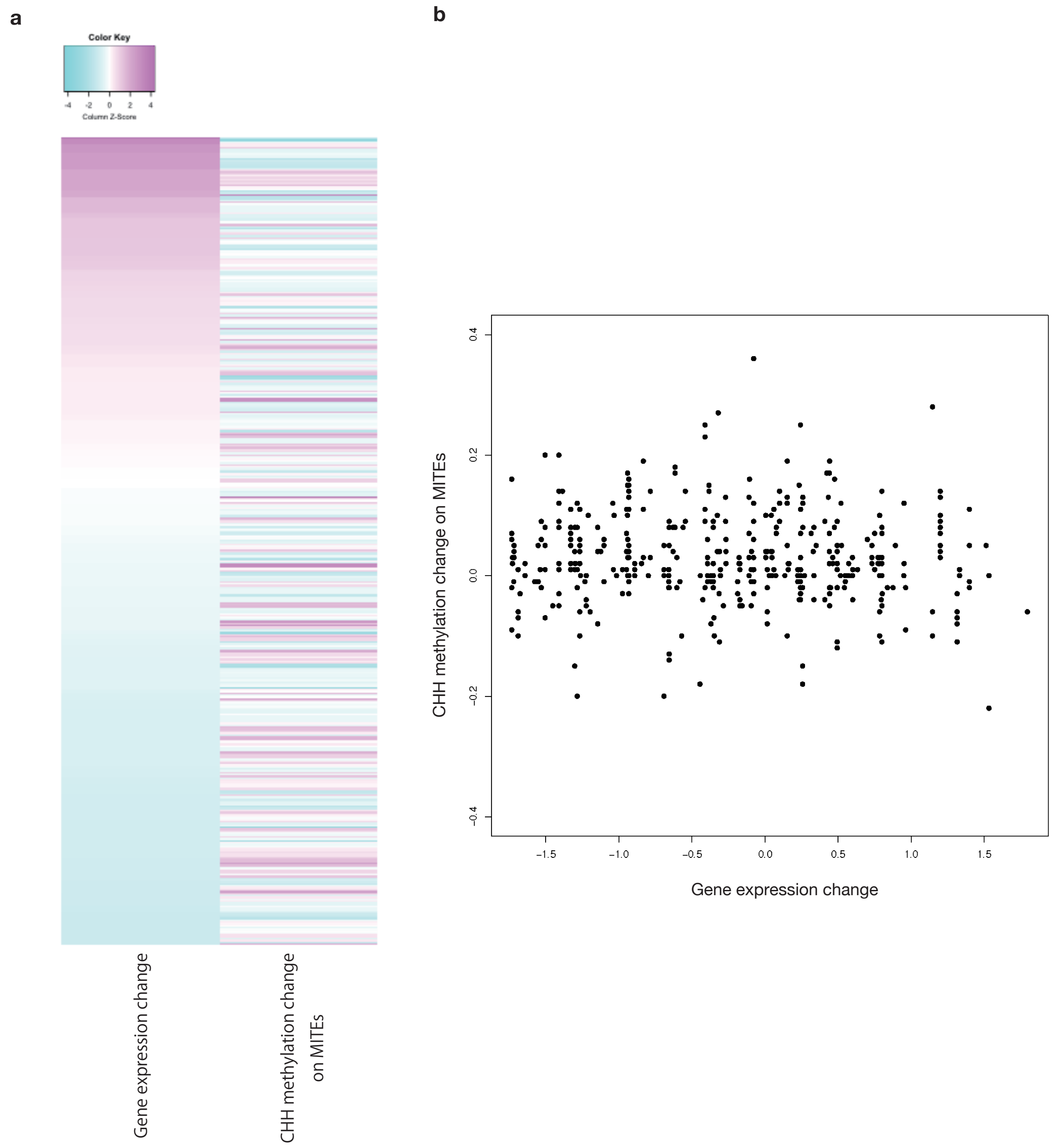

Supplementary Fig. 8. Expression level of protein-coding genes and CHH methylation levels of MITEs in the promoter region of the genes. Heatmap (a) and scatter plot (b) show the relationship between the change of the expression levels of protein-coding genes and the CHH methylation levels of MITE body in their promoter regions. CHH methylation change means the difference of CHH methylation level between reproductive SAM and vegetative SAM. Gene expression change means log2 of fold change between reproductive SAM and vegetative SAM.

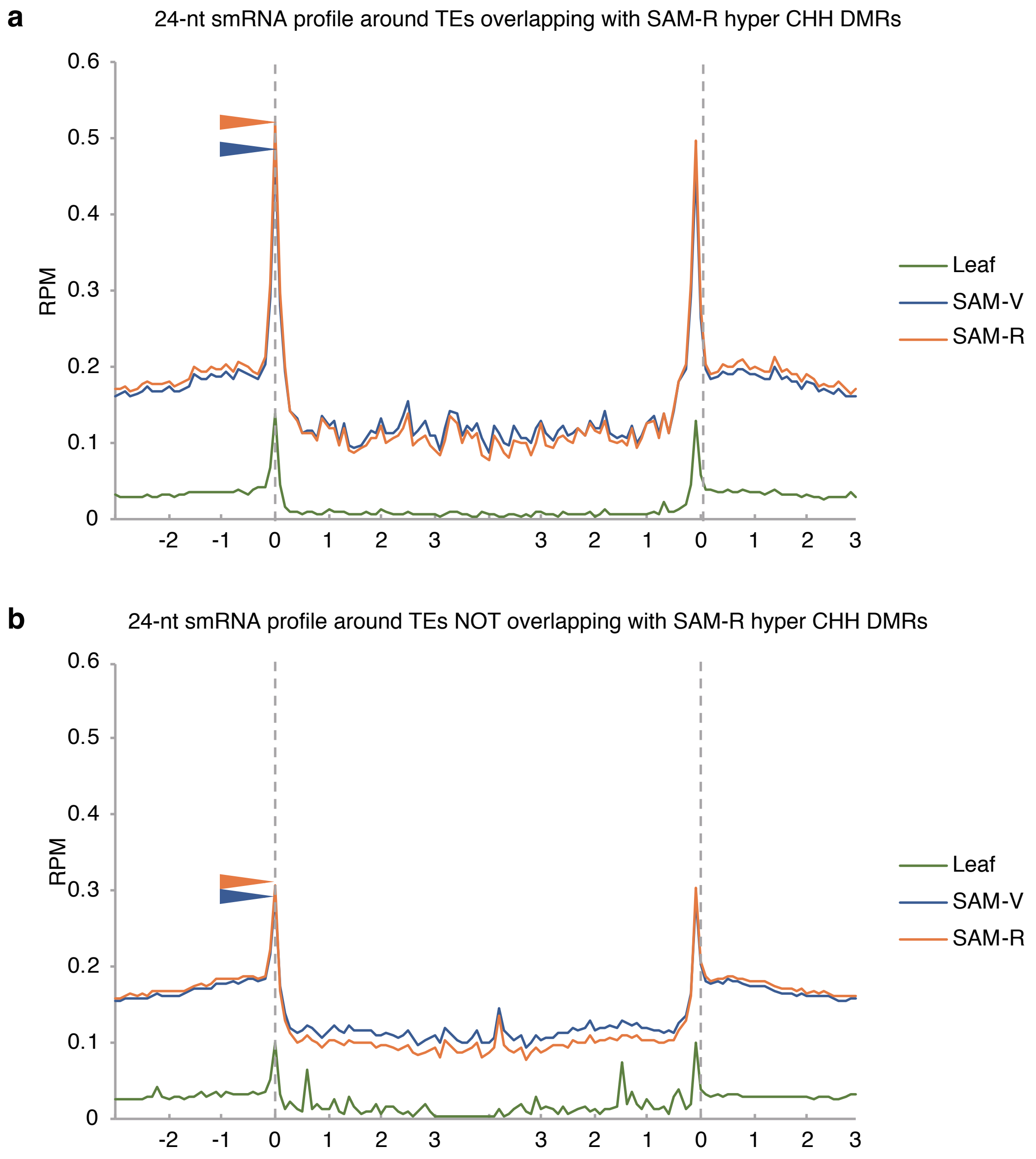

Supplementary Fig. 9. Small RNA profile along TEs overlapping with DMRs. a, b Patterns of 24-nt smRNA expression in mature leaf blade (green), vegetative SAM (blue), and reproductive SAM (red) around TEs that overlap with Rep-hyper DMRs (a) or did not overlap with Rep-hyper DMRs (b). These TEs were aligned at the 5 end or the 3 end and average RPM values for smRNAs within 100-bp windows were plotted from 3 kb away from the TEs (negative numbers) to 4 kb into the annotated regions (positive numbers). Dashed lines represent the points of alignment. Arrowheads indicate the peaks of plots for the vegetative SAM (blue) and reproductive SAM (red).

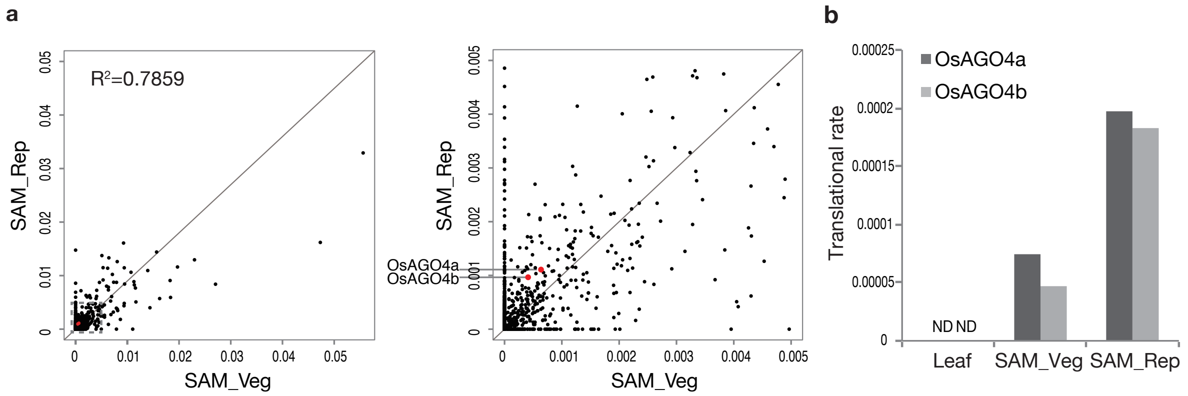

Supplementary Fig. 10. Protein levels in vegetative SAM and reproductive SAM. a, emPAI values of each protein in vegetative SAM (SAM_Veg) and reproductive SAM (SAM_Rep) are shown in a scatter plot. OsAGO4a and OsAGO4b values are shown in red. The right plot is an enlargement of the dashed box in the left plot. b, Ratio of emPAI value to normalized expression level (RPM) in RNA-seq is shown for OsAGO4a (dark gray) and OsAGO4b (light gray) to assess whether OsAGO4s are efficiently translated in the SAM. Mature leaf blade (Leaf), vegetative SAM (SAM_Veg), and reproductive SAM (SAM_Rep) are indicated. ND; not detected.

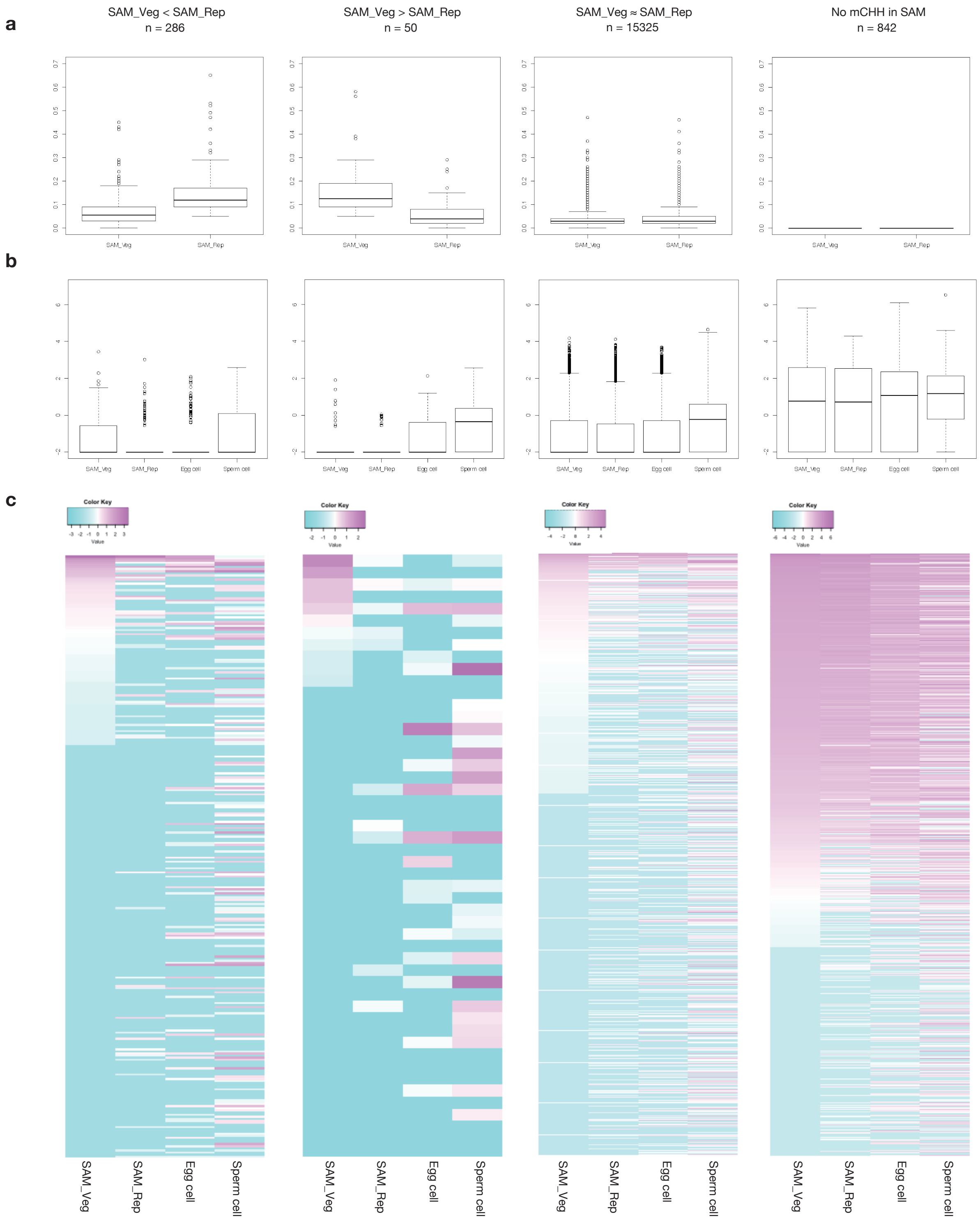

Supplementary Fig. 11. Relationship between the change of CHH methylation level in SAM and the expression level of annotated TEs in egg/sperm cell. a, Annotated TEs were divided into four groups according to the CHH methylation dynamics in SAM during the floral transition. SAM_Veg < SAM_Rep: SAM_Rep - SAM_Veg ≥ 0.05; SAM_Veg > SAM_Rep:

SAM_Rep - SAM_Veg ≤ -0.05; SAM_Veg ≈ SAM_Rep: -0.05 > SAM_Rep - SAM_Veg > 0.05 ; no mCHH in SAM: CHH methylation levels in vegetative SAM and reproductive SAM equal zero. Box plots show the CHH methylation levels of the body region of annotated TEs in each group in the vegetative SAM and reproductive SAM. **b, c,** Box plots (b) and heatmaps (c) show the expression level (log10 of normalized counts + 0.01) of annotated TEs in each group in the vegetative SAM, reproductive SAM, egg cell, and sperm cell.

a

b

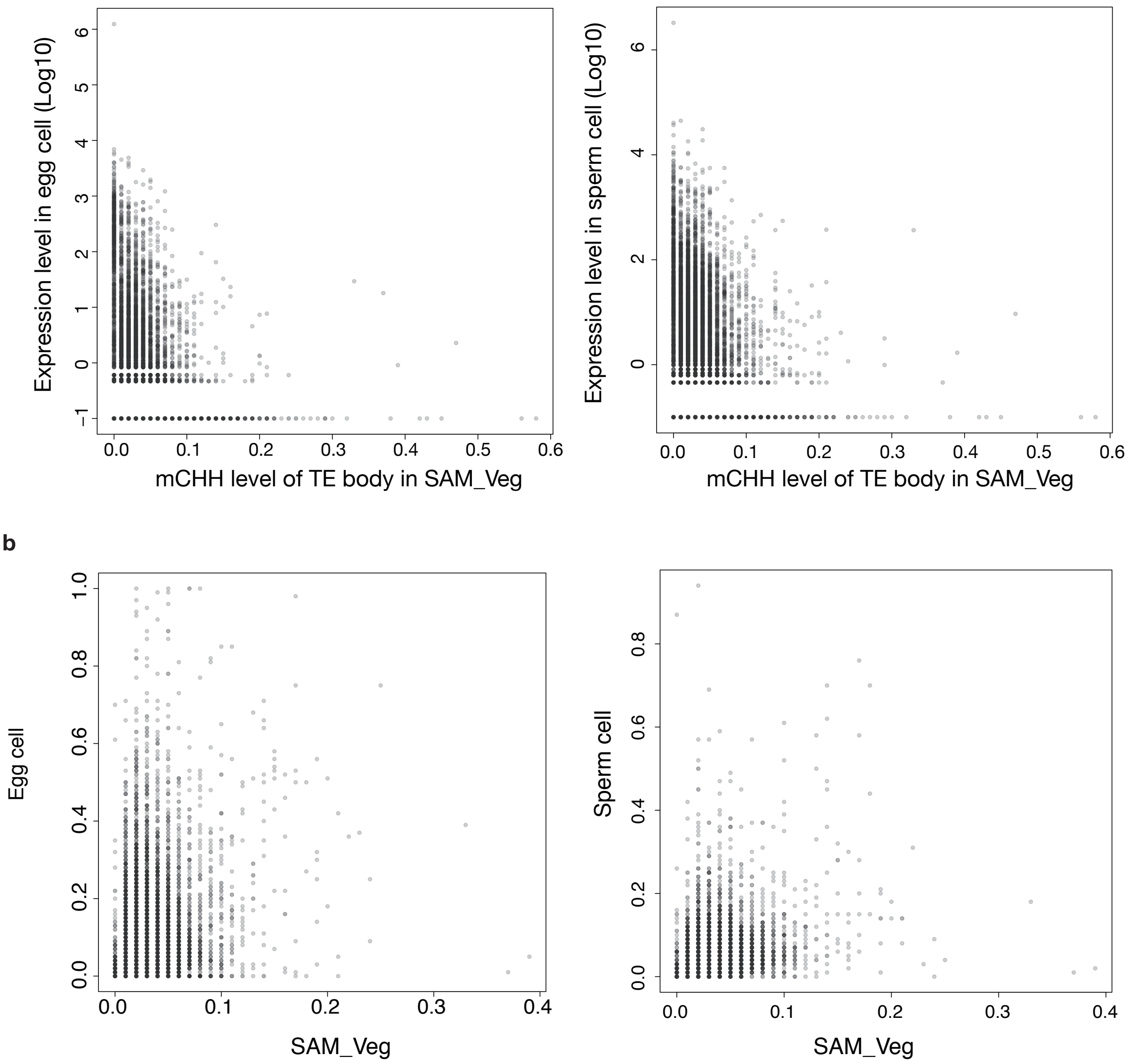

Supplementary Fig. 12. Relationship between mCHH level in vegetative SAM and the level of TE silencing in egg/sperm cell. a, The CHH methylation levels in vegetative SAM (SAM_Veg, x axis) and expression levels in egg cell (left) or sperm cell (right) (y axis) of each annotated TE are shown in a scatter plot. Expression level: log10(normalized counts + 0.01). **b,** The CHH methylation levels in vegetative SAM (SAM_Veg, x axis) and egg cell (left) or sperm cell (right) (y axis) of each annotated TE are shown in a scatter plot.

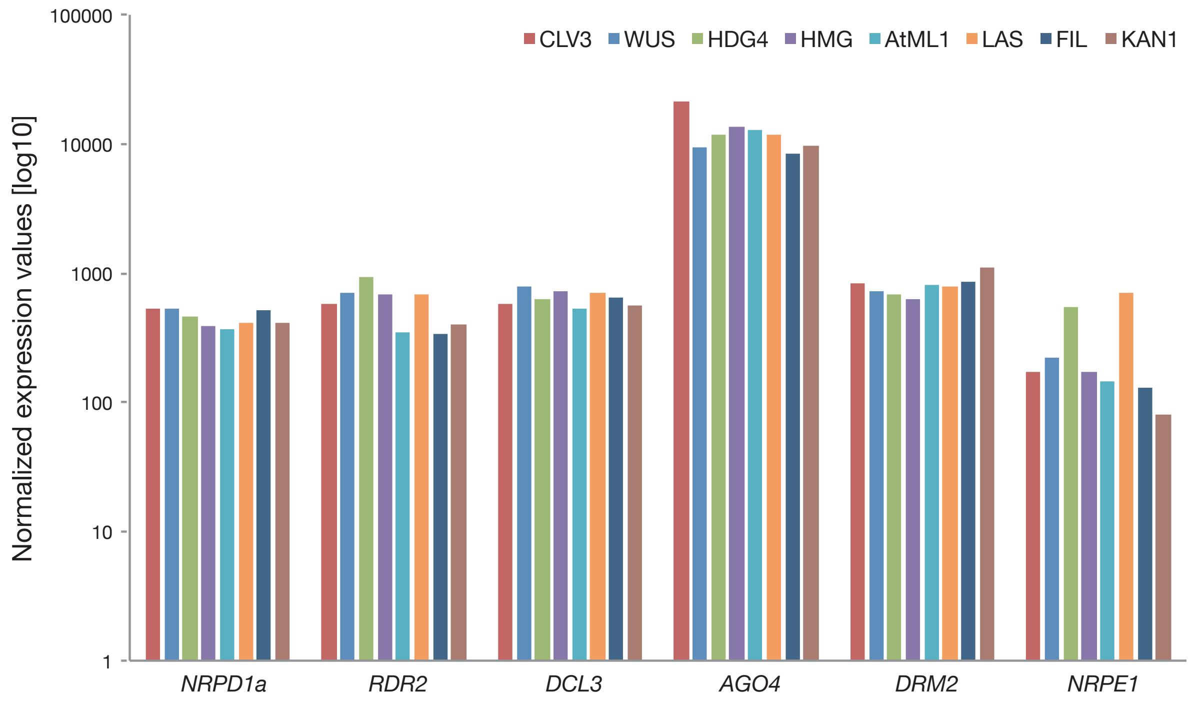

Supplementary Fig. 13. Expression of genes related to the RdDM pathway in different cell types in the Arabidopsis SAM. Expression of each gene in the cell types with the indicated markers shown. The cell type markers are CLV3 for central zone; WUS for corpus, L3 layers, and rib meristem; HDG4 for L2; HMG for L1 layer of the SAM; AtML1 for L1 of the SAM and differentiated organs; LAS for organ boundaries; FIL for organ primordia; and KAN1 for outer edges of peripheral zone.

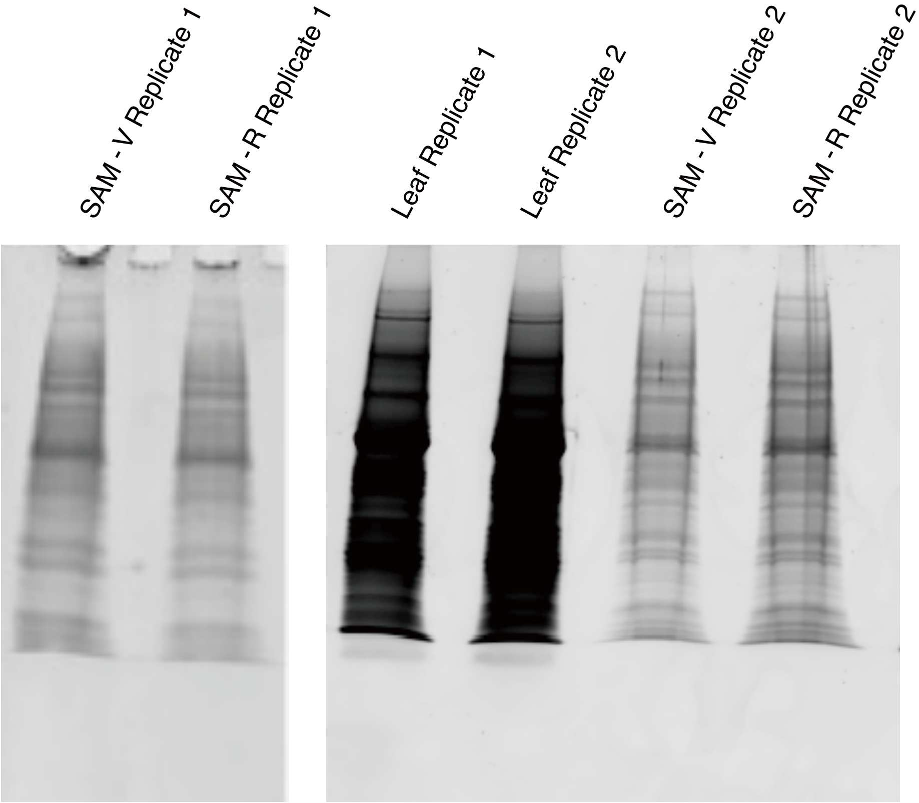

Supplementary Fig. 14. SDS-PAGE gel images of samples prepared for proteome analysis. Extracts from SAM-V, SAM-R, and leaf were separated by SDS-PAGE. After staining, the proteins in the gels were digested and subjected to MS/MS analysis, for which reason molecular weight markers were not included.
