# Supplementary material for: DNA methylation is reconfigured at the onset of reproduction in rice shoot apical meristem
Source: Nat Commun. 2020 Aug 14;11:4079. doi: 10.1038/s41467-020-17963-2 (PMC7429860; doi:10.1038/s41467-020-17963-2)
Supplement: Supplementary file 3 — Descriptions of Additional Supplementary Files [file 41467_2020_17963_MOESM3_ESM.pdf]

Descriptions of Additional Supplementary Files

Supplementary Movie 1

Description: A SAM was isolated from a rice seedling by hand dissection under microscopy.

Supplementary Data 1

Description: Normalized emPAI values of proteome analysis.

Supplementary Data 2

Description: Scores, number of peptides and sequence coverages for proteome analysis.
